# 4-Methyl­anilinium nitrate

**DOI:** 10.1107/S1600536809041488

**Published:** 2009-10-17

**Authors:** Nourredine Benali-Cherif, Houda Boussekine, Zina Boutobba, Noureddine Dadda

**Affiliations:** a Laboratoire des Structures, Propriétés et Interactions Inter Atomiques (LASPI^2^A), Centre Universitaire Abbes Laghrour-Khenchela, 40000. Khenchela, Algeria

## Abstract

The asymmetric unit of the title compound, C_7_H_10_N^+^·NO_3_^−^, consists of a 4-methyl­anilinium cation protonated at the amino group and a nitrate anion. In the crystal, anions and cations are linked through N—H⋯O and N—H⋯(O,O) hydrogen bonds, buiding a corrugated layer structure parallel to (001).

## Related literature

For related structures, see: Benali-Cherif, Kateb *et al.* (2007 ▶); Benali-Cherif, Allouche *et al.* (2007 ▶); Benali-Cherif, Boussekine *et al.* (2007 ▶); Asath Bahadur *et al.* (2007 ▶). For the bio­logical effects of toluidine exposure in man, see: Kennedy *et al.* (1984 ▶).
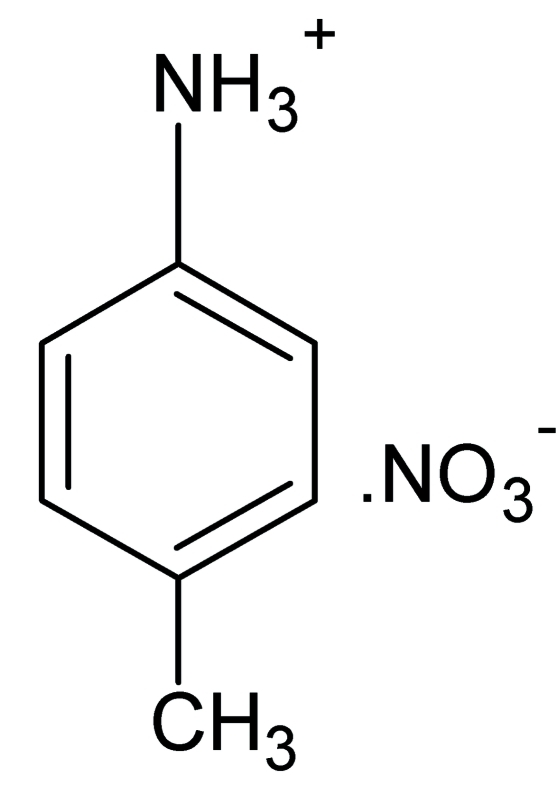


## Experimental

### 

#### Crystal data

C_7_H_10_N^+^·NO_3_^−^
*M* *_r_* = 170.17
Monoclinic, 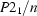

*a* = 5.6725 (9) Å
*b* = 8.5507 (8) Å
*c* = 17.621 (2) Å
β = 98.771 (2)°
*V* = 844.69 (18) Å^3^
*Z* = 4
Mo *K*α radiation
μ = 0.11 mm^−1^
*T* = 100 K
0.2 × 0.15 × 0.1 mm

#### Data collection

Nonius KappaCCD diffractometer
Absorption correction: none
24550 measured reflections
2791 independent reflections
1228 reflections with *I* > 2σ(*I*)
*R*_int_ = 0.089

#### Refinement

*R*[*F* ^2^ > 2σ(*F* ^2^)] = 0.049
*wR*(*F* ^2^) = 0.125
*S* = 0.91
2791 reflections
111 parameters
H-atom parameters constrained
Δρ_max_ = 0.29 e Å^−3^
Δρ_min_ = −0.19 e Å^−3^

### 

Data collection: *KappaCCD* (Nonius, 1998 ▶); cell refinement: *DENZO* and *SCALEPACK* (Otwinowski & Minor, 1997 ▶); data reduction: *DENZO* and *SCALEPACK*; program(s) used to solve structure: *SIR2004* (Burla *et al.*, 2005 ▶); program(s) used to refine structure: *SHELXL97* (Sheldrick, 2008 ▶); molecular graphics: *ORTEP-3* (Farrugia, 1997 ▶) and *CAMERON* (Pearce *et al.*, 2000 ▶); software used to prepare material for publication: *WinGX* (Farrugia, 1999 ▶).

## Supplementary Material

Crystal structure: contains datablocks global, I. DOI: 10.1107/S1600536809041488/dn2484sup1.cif

Structure factors: contains datablocks I. DOI: 10.1107/S1600536809041488/dn2484Isup2.hkl

Additional supplementary materials:  crystallographic information; 3D view; checkCIF report

## Figures and Tables

**Table 1:** Hydrogen-bond geometry (Å, °)

*D*—H⋯*A*	*D*—H	H⋯*A*	*D*⋯*A*	*D*—H⋯*A*
N1—H1*B*⋯O3	0.89	1.93	2.8032 (17)	167
N1—H1*A*⋯O2^i^	0.89	1.93	2.8208 (18)	177
N1—H1*C*⋯O3^ii^	0.89	2.11	2.9461 (17)	157
N1—H1*C*⋯O2^ii^	0.89	2.46	3.1726 (18)	138

Symmetry codes: (i) 
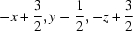
; (ii) 
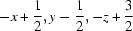
.

**Fig. 1.**
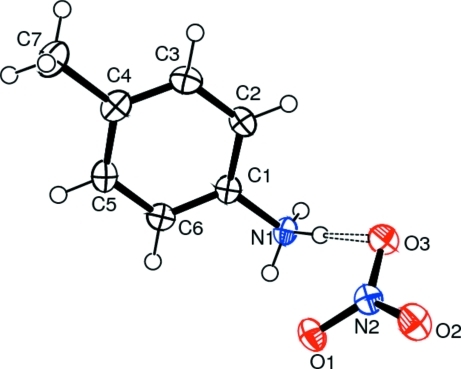
Molecular view of compound I with the atom labeling scheme. Displacement ellipsoids are drawn at the 50% probability level. H atoms are represented as small sphere of arbitray radii. Hydrogen bond is shown as dashed line.

**Fig. 2.**
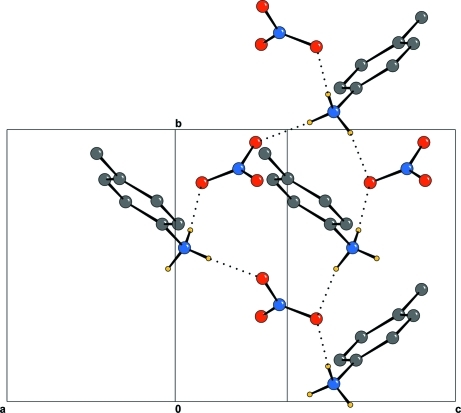
Partial packing view of the hydrogen-bonding network.

## supplementary crystallographic information

### Comment

*p*-toluidine is an organic benzene derivative with a methyl substituent and an amino group, the name is derived from toluene and aniline. Its physical appearance is that of white lustrous plates or leaflets with an amine odour. *p*-toluidine can cause anoxia (due to formation of methemoglobin) and hematuria in man. The substance irritates the eyes and the skin and may cause effects on the blood, bladder and kidneys, resulting in tissue lesions and formation of methamoglobin (Kennedy *et al.*, 1984). The crystal structure of *p*-methylanilinium nitrate, (I), was determined as part of our investigations on the structural characteristicsof organic-inorganic layered compounds and an ongoing study on *D*—*H*···A hydrogen-bonding in systems of hybrid materials including anilinium derivatives such as, 3-hydroxyanilinium hydrogensulfate (Benali-Cherif, Kateb *et al.*, 2007), *o*-methylanilinium nitrate (Benali-Cherif, Boussekine *et al.*, 2007), 2-carboxyanilinium dihydrogenphosphate (Benali-Cherif, Allouche *et al.*, 2007) and 2-carboxyanilinium nitrate (Bahadur *et al.*, 2007).

The asymmetric unit of (I) contains a monoprotonated *p*-methylanilinium cation and nitrate anion link trough N-H···O hydrogen bond (Figure 1). Intra atomic bond distances and angles confirm the monprotonation of the organic entity. There are differences in the N—O distances of nitrate anion N2—O2, N2–03 (1.260 (2) Å, 1.276 (2) Å) are longer than N2—O1 (1.232 (2) Å), this is the due that only the O2 and O3 atoms are involved in hydrogen bonds of types N—H ··· O. (Table 1). The structure of (C_7_H_10_N^+^. NO_3_^-^) is composed of cationic (C_7_H_10_N^+^) and anionic (NO_3_^-^) linked through N-H···O hydrogren bonds and building up a corrugated layers parallel to the (0 0 1) plane (Table 1, Figure 2).

### Experimental

Single crystals of the title compound are prepared by slow evaporation at room temperature of an aqueous solution of *p*-methylaniline (C_7_H_9_N) and nitric acid in the stoichiometric ratio 1:1.

### Refinement

All H atoms attached to C atoms and N atom were fixed geometrically and treated as riding with C—H = 0.96 Å (methyl) or 0.93 Å (aromatic) and N—H = 0.89 Å with U_iso_(H) = 1.2U_eq_(aromatic) or U_iso_(H) = 1.5U_eq_(methyl,N).

### Crystal data

**Table:**

C_7_H_10_N^+^·NO_3_^−^	*F*(000) = 360
*M**_r_* = 170.17	*D*_x_ = 1.338 Mg m^−^^3^
Monoclinic, *P*2_1_/*n*	Mo *K*α radiation, λ = 0.71073 Å
Hall symbol: -P 2yn	Cell parameters from 24550 reflections
*a* = 5.6725 (9) Å	θ = 2.7–31.5°
*b* = 8.5507 (8) Å	µ = 0.11 mm^−^^1^
*c* = 17.621 (2) Å	*T* = 100 K
β = 98.771 (2)°	Prism, brown
*V* = 844.69 (18) Å^3^	0.2 × 0.15 × 0.1 mm
*Z* = 4	

### Data collection

**Table:**

Nonius KappaCCD diffractometer	1228 reflections with *I* > 2σ(*I*)
Radiation source: fine-focus sealed tube	*R*_int_ = 0.089
graphite	θ_max_ = 31.5°, θ_min_ = 2.7°
ω–θ scans	*h* = −8→5
24550 measured reflections	*k* = −12→12
2791 independent reflections	*l* = −25→25

### Refinement

**Table:**

Refinement on *F*^2^	Primary atom site location: structure-invariant direct methods
Least-squares matrix: full	Secondary atom site location: difference Fourier map
*R*[*F*^2^ > 2σ(*F*^2^)] = 0.049	Hydrogen site location: inferred from neighbouring sites
*wR*(*F*^2^) = 0.125	H-atom parameters constrained
*S* = 0.91	*w* = 1/[σ^2^(*F*_o_^2^) + (0.0589*P*)^2^] where *P* = (*F*_o_^2^ + 2*F*_c_^2^)/3
2791 reflections	(Δ/σ)_max_ = 0.001
111 parameters	Δρ_max_ = 0.29 e Å^−^^3^
0 restraints	Δρ_min_ = −0.19 e Å^−^^3^

### Special details

**Table:**

Geometry. All esds (except the esd in the dihedral angle between two l.s. planes) are estimated using the full covariance matrix. The cell esds are taken into account individually in the estimation of esds in distances, angles and torsion angles; correlations between esds in cell parameters are only used when they are defined by crystal symmetry. An approximate (isotropic) treatment of cell esds is used for estimating esds involving l.s. planes.
Refinement. Refinement of *F*^2^ against ALL reflections. The weighted *R*-factor *wR* and goodness of fit *S* are based on *F*^2^, conventional *R*-factors *R* are based on *F*, with *F* set to zero for negative *F*^2^. The threshold expression of *F*^2^ > σ(*F*^2^) is used only for calculating *R*-factors(gt) *etc*. and is not relevant to the choice of reflections for refinement. *R*-factors based on *F*^2^ are statistically about twice as large as those based on *F*, and *R*- factors based on ALL data will be even larger.

### Fractional atomic coordinates and isotropic or equivalent isotropic displacement parameters (Å^2^)

**Table:**

	*x*	*y*	*z*	*U*_iso_*/*U*_eq_	
C1	0.4327 (3)	0.15225 (17)	0.90563 (9)	0.0212 (4)	
C2	0.2258 (3)	0.23416 (18)	0.91199 (10)	0.0251 (4)	
H2	0.0979	0.2342	0.8721	0.030*	
C3	0.2140 (3)	0.31609 (19)	0.97933 (10)	0.0280 (4)	
H3	0.0752	0.3705	0.9843	0.034*	
C4	0.4025 (3)	0.31936 (17)	1.03941 (10)	0.0261 (4)	
C5	0.6101 (3)	0.23714 (18)	1.03075 (9)	0.0265 (4)	
H5	0.7396	0.2386	1.0701	0.032*	
C6	0.6249 (3)	0.15283 (17)	0.96364 (9)	0.0239 (4)	
H6	0.7629	0.0979	0.9583	0.029*	
C7	0.3867 (4)	0.4121 (2)	1.11156 (10)	0.0347 (5)	
H7A	0.4333	0.5184	1.1044	0.052*	
H7B	0.4911	0.3670	1.1539	0.052*	
H7C	0.2257	0.4099	1.1222	0.052*	
N1	0.4466 (2)	0.06544 (14)	0.83447 (7)	0.0236 (3)	
H1A	0.5927	0.0263	0.8361	0.035*	
H1B	0.4147	0.1296	0.7945	0.035*	
H1C	0.3409	−0.0122	0.8298	0.035*	
N2	0.5736 (3)	0.35591 (15)	0.71667 (8)	0.0249 (3)	
O1	0.7537 (2)	0.31274 (13)	0.75970 (7)	0.0311 (3)	
O2	0.5852 (2)	0.45260 (13)	0.66347 (7)	0.0339 (3)	
O3	0.3676 (2)	0.30432 (13)	0.72457 (7)	0.0295 (3)	

### Atomic displacement parameters (Å^2^)

**Table:**

	*U*^11^	*U*^22^	*U*^33^	*U*^12^	*U*^13^	*U*^23^
C1	0.0282 (9)	0.0117 (7)	0.0233 (9)	−0.0041 (7)	0.0031 (7)	0.0006 (6)
C2	0.0245 (9)	0.0180 (8)	0.0312 (10)	−0.0018 (7)	−0.0012 (7)	0.0002 (7)
C3	0.0290 (10)	0.0182 (8)	0.0373 (11)	0.0018 (7)	0.0066 (8)	−0.0007 (7)
C4	0.0381 (10)	0.0130 (7)	0.0285 (10)	−0.0053 (7)	0.0095 (8)	0.0016 (7)
C5	0.0333 (10)	0.0207 (8)	0.0241 (10)	−0.0042 (7)	0.0001 (8)	0.0029 (7)
C6	0.0261 (9)	0.0167 (8)	0.0291 (10)	0.0000 (7)	0.0051 (7)	0.0036 (7)
C7	0.0524 (12)	0.0222 (8)	0.0305 (11)	−0.0023 (8)	0.0097 (9)	−0.0023 (7)
N1	0.0277 (8)	0.0166 (6)	0.0260 (8)	−0.0014 (6)	0.0022 (6)	0.0002 (6)
N2	0.0290 (8)	0.0169 (7)	0.0279 (8)	0.0011 (6)	0.0018 (7)	−0.0022 (6)
O1	0.0276 (7)	0.0305 (7)	0.0326 (7)	0.0051 (6)	−0.0038 (6)	0.0023 (6)
O2	0.0354 (7)	0.0252 (6)	0.0397 (8)	−0.0020 (6)	0.0008 (6)	0.0139 (6)
O3	0.0277 (7)	0.0267 (6)	0.0338 (7)	−0.0015 (5)	0.0041 (5)	0.0052 (5)

### Geometric parameters (Å, °)

**Table:**

C1—C6	1.376 (2)	C6—H6	0.9300
C1—C2	1.386 (2)	C7—H7A	0.9600
C1—N1	1.470 (2)	C7—H7B	0.9600
C2—C3	1.388 (2)	C7—H7C	0.9600
C2—H2	0.9300	N1—H1A	0.8900
C3—C4	1.385 (2)	N1—H1B	0.8900
C3—H3	0.9300	N1—H1C	0.8900
C4—C5	1.399 (2)	N2—O1	1.2325 (17)
C4—C7	1.513 (2)	N2—O2	1.2592 (16)
C5—C6	1.398 (2)	N2—O3	1.2759 (17)
C5—H5	0.9300		
			
C6—C1—C2	121.53 (15)	C5—C6—H6	120.4
C6—C1—N1	119.74 (14)	C4—C7—H7A	109.5
C2—C1—N1	118.73 (14)	C4—C7—H7B	109.5
C1—C2—C3	118.41 (16)	H7A—C7—H7B	109.5
C1—C2—H2	120.8	C4—C7—H7C	109.5
C3—C2—H2	120.8	H7A—C7—H7C	109.5
C4—C3—C2	122.09 (16)	H7B—C7—H7C	109.5
C4—C3—H3	119.0	C1—N1—H1A	109.5
C2—C3—H3	119.0	C1—N1—H1B	109.5
C3—C4—C5	118.08 (16)	H1A—N1—H1B	109.5
C3—C4—C7	121.01 (16)	C1—N1—H1C	109.5
C5—C4—C7	120.90 (17)	H1A—N1—H1C	109.5
C6—C5—C4	120.75 (16)	H1B—N1—H1C	109.5
C6—C5—H5	119.6	O1—N2—O2	121.47 (14)
C4—C5—H5	119.6	O1—N2—O3	121.07 (14)
C1—C6—C5	119.14 (16)	O2—N2—O3	117.45 (14)
C1—C6—H6	120.4		

### Hydrogen-bond geometry (Å, °)

**Table:**

*D*—H···*A*	*D*—H	H···*A*	*D*···*A*	*D*—H···*A*
N1—H1B···O3	0.89	1.93	2.8032 (17)	167
N1—H1A···O2^i^	0.89	1.93	2.8208 (18)	177
N1—H1C···O3^ii^	0.89	2.11	2.9461 (17)	157
N1—H1C···O2^ii^	0.89	2.46	3.1726 (18)	138

Symmetry codes: (i) −*x*+3/2, *y*−1/2, −*z*+3/2; (ii) −*x*+1/2, *y*−1/2, −*z*+3/2.
